# Editorial: Integration of advanced technologies in orchard management

**DOI:** 10.3389/fpls.2026.1801415

**Published:** 2026-05-26

**Authors:** Edney Leandro da Vitoria, Zhenghong Yu

**Affiliations:** 1 Departament of Agricultural and Biological Science, Federal University Espirito Santo, São Mateus, Brazil; 2 Guandong Polytchnic of Science ant Technology, Huazhong University of Science and Technology, Guandong, China

**Keywords:** artificial intelligence, IoT, orchards, pesticides, phytoprotection, precision agriculture, UAVs

Orchard management faces increasing pressure to align productivity with environmental sustainability in order to modernize processes, agricultural operations and management. Besides that, there is the need to minimize contamination from agrochemicals, optimize resources, and ensure food safety that drive the transition from traditional practices to technology-based models. In this context, sustainable and smart phytoprotection emerges as an essential paradigm, which integrates information and communication science progress with consolidated agronomic knowledge. However, the digital revolution in agriculture is not merely a matter of adopting new tools, but it represents a fundamental change in understanding, monitoring, and interacting with agricultural systems to enable real-time and data-driven decision-making.

**Figure 1:**
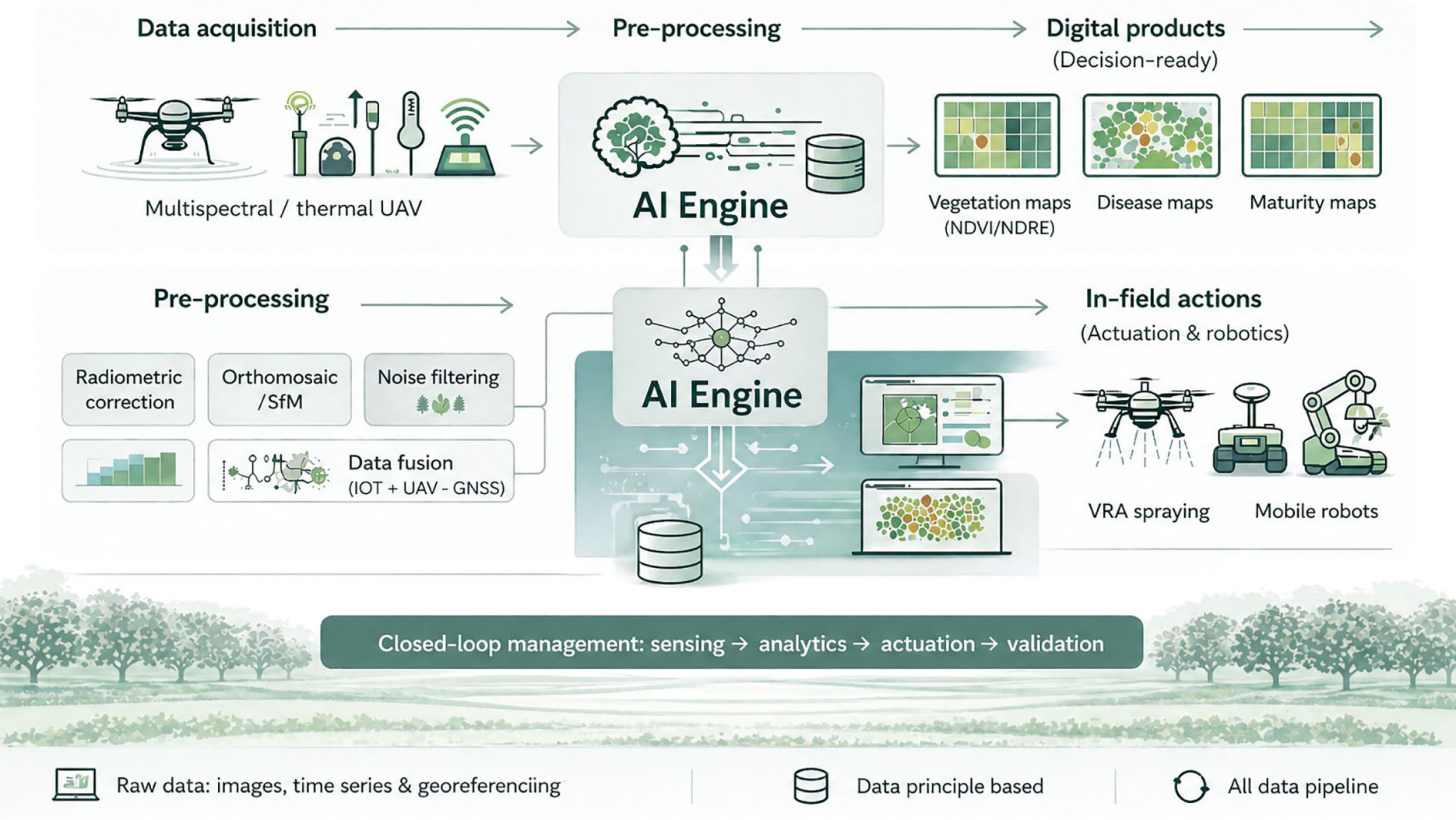
Advanced technology integrated architecture in orchard management.

In other words, and as shown in **Figure 1** below, the beforementioned integrated approach involves data flow from collection via remote and IoT sensors to artificial intelligence processing and data analysis, and then in practical applications of monitoring, detection, precision spraying, and automation. As it can be also seen, the bidirectional arrows mean continuous feedback that enables system optimization and learning.

Thanks to this scenario, the paper - Integration of Advanced Technologies in Orchard Management - was conceived with the aim of gathering and disseminating the latest development that is at the forefront of this transformation. Nine articles were selected to provide a comprehensive overview of the innovations that are redefining the way orchards are monitored, managed and protected, from the input precise application to selective harvesting. These studies, developed by researchers from leading institutions in different continents, reflect the truly global nature of technological transformation and the growing convergence of efforts to address common challenges in contemporary fruit growing.

As revealed by the contributions of those articles, the transformation can be divided into three interconnected research frontiers: (1) computer view and artificial intelligence for monitoring and diagnosis; (2) precision spraying technologies; and (3) robotics and remote sensing for automation and data collection. Although distinct in their technical approaches, these areas share a common goal: turning raw data into actionable intelligence that allows producers to make more precise, efficient, and environmentally responsible decisions.

Firstly, in the field of computer view and artificial intelligence, the chosen articles demonstrate remarkable progress in the ability to identify and classify targets of interest in complex scenarios. The studies cover from the detection of citrus varieties in real time, using enhanced models such as YOLOv7 (Deng et al.) to the identification of sunburn on citrus fruits with YOLOv8-Scm (Cong et al.). The application extends to pomegranate maturity assessment by the development of the DFMA-DETR algorithm (Huang et al.), and the efficient detection of flowers of the genus Alstroemeria (Feng et al.), which illustrates the versatility of these tools for different crops and purposes. Hence, the continuous improvement of algorithms, particularly through deep learning techniques, allows not only greater accuracy in detection, but also greater robustness in variable conditions of illumination, occlusion and environmental complexity, which are intrinsic characteristics in real agricultural scenarios.

Secondly, the optimization of spraying technologies is another important highlight in this paper, specifically about the increase in efficiency and environmental impact reduction when applying pesticides in orchards. For instance, an in-depth study on gas-liquid flow dynamics in multi-duct sprayers, based on computational fluid dynamics (CFD), offers new perspectives for the design of more effective equipment (Li et al.). Complementarily, the research on tips hydraulic air-assisted spraying demonstrates significant improvement in droplet deposition and drift reduction (Ou et al.). Furthermore, the integration of Global Navigation Satellite Systems (GNSS) with leaf area density sensors for variable rate spraying represents a significant advance towards localized and intelligent application of inputs (Zhao et al.). These innovations are particularly relevant considering the increasing environmental regulations that limit the use of pesticides and require greater efficiency in their application, turning precision spraying into an economic and environmental necessity.

Finally, robotics and remote sensing consolidate as indispensable tools for automation and large-scale data collection. For example, the development of an autonomous navigation method for mobile robots, based on the optimization of 3D point clouds, paves the way for the execution of complex tasks without continuous human intervention (Li et al.). Also, the use of unmanned aerial vehicles (UAVs) equipped with remote sensors and AI models, such as YOLOv8, detect the maturation status of lychees, which illustrates the potential of technology fusion to optimize harvest timing and maximize production value (Liang et al.). The ability to collect spatial and temporal high-resolution data, combined with intelligent processing, allows producers to monitor plant health and development, along with several details that were impossible before, turning orchard management from an experience-based activity into a data-driven approach.

All the articles selected in this paper not only highlight the state of the art in technological management of orchards, but they also signal a promising future in which precision agriculture and intelligent automation become the standard. The remaining challenges, such as the integration of data from multiple sources, cost reduction and scalability of solutions, will continue to provide valuable opportunities for further research. Particularly important is the development of integration models that allow communication between different systems and platforms, as well as the creation of viable economic models that make these technologies accessible to producers of different scales. It is hoped this paper inspires new studies that deepen the mentioned critical issues and others that may accelerate the transition to truly smart and sustainable orchards.

To sum up, outstanding contributions were made by the authors and reviewers to this important area of research.

